# Pain Catastrophizing: How Far Have We Come

**DOI:** 10.3390/neurolint16030036

**Published:** 2024-04-26

**Authors:** Katarina Simic, Boris Savic, Nebojsa Nick Knezevic

**Affiliations:** 1 Department of Anesthesiology, Advocate Illinois Masonic Medical Center, Chicago, IL 60657, USA; simickatarina246@gmail.com (K.S.); borissavic421@icloud.com (B.S.); 2 Department of Anesthesiology, University of Illinois, Chicago, IL 60612, USA; 3 Department of Surgery, University of Illinois, Chicago, IL 60612, USA

**Keywords:** pain catastrophizing, chronic pain, depression, anxiety, cognitive-behavioral therapy

## Abstract

The perception of pain is strongly influenced by various social, emotional, and cognitive factors. A psychological variable which has consistently been shown to exert its influence on pain is a cognitive process referred to as pain catastrophizing. Numerous studies have found it to be a strong predictor of pain intensity and disability across different clinical populations. It signifies a maladaptive response to pain marked by an exaggerated negative assessment, magnification of symptoms related to pain, and, in general, a tendency to experience marked pain-related worry, as well as experiencing feelings of helplessness when it comes to dealing with pain. Pain catastrophizing has been correlated to many adverse pain-related outcomes, including poor treatment response, unsatisfactory quality of life, and high disability related to both acute and chronic pain. Furthermore, there has been consistent evidence in support of a correlation between pain catastrophizing and mental health disorders, such as anxiety and depression. In this review, we aim to provide a comprehensive overview of the current state of knowledge regarding pain catastrophizing, with special emphasis on its clinical significance, and emerging treatment modalities which target it.

## 1. Introduction

Pain is “an unpleasant sensory and emotional experience associated with, or resembling that associated with, actual or potential tissue damage” [1]. In order to gain a complete understanding of the experience of pain, it is most commonly assessed in a biopsychosocial context [2], as the most broad, thorough approach, needed for a comprehensive analysis of such a complex subject [3]. The intensity, severity, and perception of pain are strongly influenced not only by biological factors, but by emotional and social variables as well [4]. A psychological factor which has consistently been shown to exert its influence on pain is a cognitive process referred to as pain catastrophizing. Numerous studies in the last thirty years have found it to be a strong predictor of pain intensity and disability across different clinical populations [5]. While it has been defined in many ways, most definitions of pain catastrophizing include an emphasis on the role of magnification of the threat value of pain, and the feeling of helplessness related to the experience of pain. Individuals that exhibit a high level of pain catastrophizing usually also have a tendency to attribute a threatening meaning to the experience of pain [6]. Another common trend is the feeling of lacking control over the experience of pain [7]. There is commonly an overstated negative reaction to pain in both acute and chronic settings, as well as in terms of pain anticipation [8].

Use of the term “pain catastrophizing” has recently been much debated [9]; critics of the term have been calling for a need to rename it, and in doing so have argued that its use promotes a negative bias towards individuals with a high level of pain catastrophizing, as well as the fact that it adds to the systemic mismanagement of patients with chronic pain [10]. Others have advocated that the mere term has little to do with those issues as they are far more complex and need a deeper, more structural approach to solve [9]. Even though the term and its use may evolve in the future, pain catastrophizing as a concept has been well established in the past and includes different cognitive, emotional, and behavioral mechanisms that profoundly affect pain perception, as well as its severity, intensity, and the possibility of treatment [11].

Over the past few decades, research into pain catastrophizing has been increasingly popularized due to the recognition of the crucial role it plays in modifying the experiences of pain and its management [4]. Gaining a deeper understanding of pain catastrophizing would help further our knowledge of acute and chronic pain conditions, as well as illuminate interventions most effective in alleviating distress and improving quality of life for affected individuals. Pain catastrophizing has also been strongly correlated to many disorders such as acute pain [12,13,14,15], chronic pain syndromes [16,17,18], malignancies [19], autoimmune disorders [20,21], and infectious diseases [22], as well as with a variety of psychiatric disorders such as generalized anxiety disorder, depression, and different psychosomatic conditions [9,23,24,25]. Even though pain is not considered to be a primary component of these disorders, or is not considered to be a component at all, the correlation between them and pain catastrophizing has been consistently strong, independent of other variables [9,24,25]. Therefore, cognitive, emotional, and other mechanisms lying at the core of the concept of pain catastrophizing are valuable topics of research, as they present potential therapy objectives which could be beneficial in the management of a large number of disorders.

In this review, we aim to provide a comprehensive overview of the current state of knowledge regarding pain catastrophizing. We will begin by elaborating on the term and conceptualization of pain catastrophizing, as well as the tools used for its assessment, and we will later discuss key theoretical frameworks that have guided research in this area. Subsequently, we will explore the evidence linking pain catastrophizing to various aspects of the pain experience, including pain intensity, emotional distress, disability, and treatment outcomes. Furthermore, this review will evaluate the clinical significance of pain catastrophizing, discussing its importance in both acute and chronic pain settings. We will also consider the complex relationship of pain catastrophizing and mental health, with an emphasis on the importance of integration of psychosocial factors into pain management approaches.

Overall, by overviewing existing research findings and identifying possibilities for future investigation, this review aims to enhance our understanding of pain catastrophizing and its implications for clinical practice and research. By understanding the complexities of the relationship between psychological processes and pain perception, we can advance towards a more holistic, effective approach to pain management, ultimately improving the well-being of individuals living with pain.

## 2. Theoretical Foundations of Pain Catastrophizing

The first use of the term catastrophizing has largely been accredited to Albert Ellis in 1962, where he described it as an “irrationally negative forecast of anticipated events” [26]. Later, it was described more closely by Beck and Emery as “dwelling on the worst possible outcomes of any situation in which there is a possibility of an unpleasant outcome” [27]. Even though the term was originally coined as a name for a broad cognitive process unrelated to pain specifically, it has since been most associated with the experience of pain, and almost the entire body of research mentioning catastrophizing is in terms of its relationship with pain [9]. Since pain catastrophizing is such an extensively observed concept, it has been defined and redefined many times. However, the most widely used definition is the one by Sullivan and colleagues, where pain catastrophizing is defined as “an exaggerated negative mental set brought to bear during actual or anticipated pain experience” [28].

**Figure 1:**
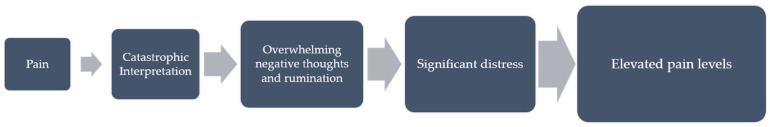
Conceptualization of pain catastrophizing according to cognitive-behavioral theory.

**Figure 2:**
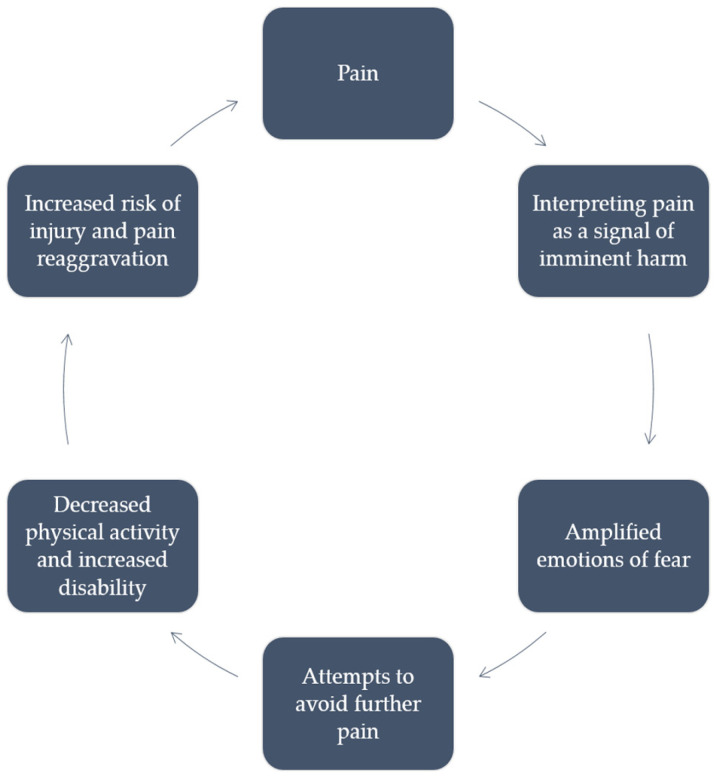
Conceptualization of pain catastrophizing according to fear-avoidance model.

What is common for most of the definitions of pain catastrophizing is the emphasis they put on a maladaptive response to pain marked by an exaggerated negative assessment, magnification of symptoms related to pain, and, in general, the tendency to experience marked pain-related worry, as well as experiencing feelings of helplessness when it comes to dealing with pain. Several theoretical frameworks have been offered in hopes of explaining the nature of pain catastrophizing. Among them, particularly valuable have been the cognitive-behavioral theory [27,29] and the fear-avoidance theory [30,31] (see Figure 1 and Figure 2).

The cognitive-behavioral theory [27] postulates that pain catastrophizing is a dysfunctional cognitive process, marked by selective attention to painful stimuli, interpretation of pain sensations as catastrophic, and maladaptive coping mechanisms. According to this theory, individuals prone to pain catastrophizing tend to amplify the perceived threat of pain which further undermines their ability to effectively cope with it [29]. In doing so, the importance of pain sensations is magnified and the negative thoughts of pain are experienced over and over again, in a ruminating manner [32], resulting in significant distress, elevated pain levels, and lessening of therapeutic effects, especially if they do not include any cognitively-oriented interventions [33,34].

On the other hand, according to the fear-avoidance model, individuals with high levels of pain catastrophizing are more likely to experience pain as a signal of imminent harm, which acts as a precipitating factor for amplified emotions of fear [35]. Afterwards, in an exaggerated, but ultimately futile attempt to avoid pain, they adopt behaviors that they perceive as preventing any such harm from occurring [35,36]. However, since these behaviors are prevalently maladaptive, they also lead to decreased levels of physical activity, which in turn increases their risk of many chronic illnesses, leads to greater disability, lessens their quality of life, and paradoxically can eventually lead to an even greater severity in pain [31,37]. A 2022 meta-analysis conducted by Rogers and Farris [3] provided further support for the fear-avoidance model. Specifically, they included a significant number of studies (n = 335) and found that, in individuals suffering from various pain conditions, there is a medium to large association between pain catastrophizing, fear of pain, and pain vigilance and pain-related negative affect, anxiety, depression, pain intensity, and disability. Another important study with possible clinical implications is a longitudinal study conducted in 2020 by Slepian et al. [38]. It demonstrated the efficacy of pain prediction using the fear-avoidance model, especially when incorporating pain resiliency. This could prove to be especially significant in future research on clinical applications of pain catastrophizing measuring scales, and their role in outlining medical treatment of pain conditions.

Research into the molecular foundations and biomarkers associated with pain catastrophizing is still evolving; however, there has been a rising number of neurobiological mechanisms implicated in pain catastrophizing. For instance, serotonin plays a crucial role in modulating mood, stress responses, and pain perception, and has been extensively researched in association with depression and anxiety [39]. Recently, serotonin has also been connected to pain catastrophizing, since a polymorphism in serotonin receptor 3B was found to be associated with pain catastrophizing [40]. Another possible molecular mechanism of pain catastrophizing may include the process of neuroinflammation. In support of this claim, there have been several recent studies [41,42] that examined a correlation between levels of pain catastrophizing and inflammatory markers. One such study by Giordano et al. (2024) [43] found that there is a correlation between GM-CSF, TGF-α, and MIP-1β and pain catastrophizing. Interestingly, GM-CSF was also found to correlate with pain occurrence in animal models, indicating it as a biomarker of induction of pain [44]. Genetic factors have also emerged as an important feature in the neurobiology of pain catastrophizing, as they also contribute to individual differences in pain processing and coping mechanisms. One such polymorphism that was found to increase susceptibility to pain catastrophizing is the Val66Met polymorphism of the brain-derived neurotrophic factor in fibromyalgia [45]. Identifying reliable biomarkers for pain catastrophizing could facilitate personalized treatment approaches aimed at mitigating its impact on pain perception and functional outcomes. However, further research is needed to validate these biomarkers and elucidate their relationships with pain catastrophizing.

In recent years, researchers have explored the intriguing connection between pain catastrophizing and social pain in animals. Studies in animal models have revealed that social stressors can exacerbate pain responses, mimicking aspects of pain catastrophizing seen in humans. A study by Arora et al. (2018) [46] found that rats that were exposed to social stress before surgery, had increased microglial activation and neuronal sensitization, as well as an overall slowed recovery after surgery. Similarly, another study conducted on a rat population found that hyperactivity in the Anterior Cingulate Cortex was correlated to catastrophizing behavior and that by inhibiting this hyperactivity the level of pain catastrophizing may be reduced [47]. These findings suggest that social factors can modify even the molecular pain processing mechanisms, and contribute to the development or exacerbation of maladaptive pain behaviors.

There has also been consistent evidence of an association between pain catastrophizing and several key brain areas. The aforementioned Anterior Cingulate Cortex which is involved in emotional processing and pain modulation, most likely plays a central role, and heightened activity in the Anterior Cingulate Cortex has been reliably observed in individuals who catastrophize pain [48]. The Prefrontal Cortex, particularly the Dorsolateral Prefrontal Cortex and the Ventromedial Prefrontal Cortex, are also implicated [48]. These regions are associated with cognitive appraisal and emotional regulation, and their altered function may likely contribute to the exaggerated interpretation of pain seen in pain catastrophizing. A meta-analysis by Malfliet et al. (2017) [49] determined that there is a correlation between pain catastrophizing and a decrease in gray matter in the Dorsolateral Prefrontal Cortex in patients with irritable bowel syndrome [50], as well as in chronic pain syndromes [51]. Furthermore, their findings describe a decrease in pain catastrophizing after interventions with cognitive-behavioral therapy, which was also correlated with an increase in gray matter in the left Dorsolateral Prefrontal Cortex, the right Posterior Parietal Cortex, the Inferior Frontal Gyrus, and the bilateral Anterior Cingulate Cortex/Medial Prefrontal Cortex [51]. These findings are especially significant due to their implications in possible new therapeutic approaches when it comes to pain catastrophizing, as well as chronic pain syndromes. Furthermore, the Insula, a brain region involved in interoception and subjective awareness of bodily states, shows increased activation in individuals with high levels of pain catastrophizing [48,49]. These findings are in line with other studies that suggest heightened awareness and sensitivity to bodily sensations may contribute to the maladaptive pain processing characteristic of catastrophizing [23]. Pain catastrophizing has also been associated with changes in resting-state functional connectivity [52]. These neural correlates may prove to be valuable biomarkers for pain catastrophizing, and further research should be conducted in support of these findings, to illuminate the processes underlying it, as well as forming personalized treatment approaches.

## 3. Assessment Tools for Pain Catastrophizing

There is a growing need to identify individuals whose pain-related outcomes may be influenced by maladaptive cognitive processes. Their early identification is important because, in doing so, personalized therapeutic approaches can be designed with specific goals and strategies in mind for each patient. For those reasons, different measuring instruments of pain catastrophizing have been developed, and the most widely used is the Pain Catastrophizing Scale (PCS) [53].

PCS is a 13-item self-report questionnaire, comprising three subscales: rumination, magnification, and helplessness. Respondents report on the extent to which they experience each thought or feeling when experiencing pain on a 5-point Likert scale, ranging from 0 (not at all) to 4 (all the time). In order to expedite the assessment, abbreviated versions of PCS have also been designed, such as the PCS-4 [54] and PCS-6 [55] which have four and six items, respectively. They were constructed with the purpose of being used in clinical settings where time for assessments can be limited; however, their brevity makes them significantly unreliable in terms of an unacceptably large standard errors of measurement [56]. Instruments for measuring pain catastrophizing in specific populations have also been designed, such as the Pain Catastrophizing Scale for Children (PCS-C) [57]. Another notable measuring instrument is the Coping Strategies Questionnaire (CSQ) [58] which evaluates a broader range of pain coping strategies, including catastrophizing. The PCS has high internal consistency, test–retest reliability, and construct validity across various populations; however, there is still some ongoing debate when it comes to measuring pain catastrophizing [56].

The experience of pain is complex, which makes it inherently difficult to definitively assess. In addition, by using the mere term pain catastrophizing in a clinical setting, there is room for a potentially negative bias against individuals who exhibit high levels of pain catastrophizing. Due to the subjective nature of pain perception, patients who score high on scales measuring pain catastrophizing could be wrongfully perceived as purposefully exaggerating their symptoms, or in other ways be held responsible for their conditions, which could lead to inadequate management [6]. This may be somewhat mitigated by substituting it with other terms such as pain-related worrying or distress [59]. However, even that would not completely resolve the negative perception of pain catastrophizing among medical professionals. Specifically, there is a significant lack of adequate training and education of medical professionals when it comes to understanding pain and pain management [60]. Therefore, most medical professionals who use pain catastrophizing measuring scales in clinical settings are not adequately trained to do so, nor are they proficient in interpreting the results, leading to an elevated risk of mismanagement of pain conditions [9]. This is a deep, systemic problem concerning health care professionals, and should be a point of discussion in further academic research of assessing pain catastrophizing.

## 4. Prevalence, Demographics, and Cultural Variations of Pain Catastrophizing

Pain catastrophizing as a phenomenon has been universally observed in different populations [61]. The prevalence varies depending on the assessment measures used, but Brouwer et al. (2020) [62] demonstrated that severe pain catastrophizing was reported in 39% of patients suffering from chronic pain.

Demographic factors such as age, gender, and socioeconomic status have been consistently shown to correlate with pain catastrophizing [63]. Research has shown that younger individuals and females are more likely to exhibit catastrophic thinking about pain than older individuals and males [6]. This difference could possibly be attributed to restrictive societal norms when it comes to emotional expression, which are commonly more prevalent in older males. On the other hand, individuals from lower socioeconomic backgrounds have shown higher levels of pain catastrophizing [63]. This is possibly due to increased exposure to stressors, work environments that are commonly more injury prone, and/or require higher levels of physical exertion. Other possible influencing factors could be the exceedingly high costs of medical treatment and limited access to resources for coping with pain, which in turn lead to a fear of financial debt, as well as of loss of income. This may result in higher rates of adverse effects in terms of disability, lower quality of life, and pain-related distress.

Some studies have reported cultural differences in the prevalence and manifestation of pain catastrophizing. For example, studies examining pain catastrophizing in Latino populations have highlighted the role of familialism and collectivist values in health-related outcomes, as well as the importance of social support networks which can function as a protective factor for depression and other stress-related affective disorders [64]. However, these findings have been inconsistent [65] and can hardly be generalized as they are strongly dependent on multiple factors, and no definitive conclusions can be derived. Nonetheless, in an everyday clinical setting, a consideration of each individual’s cultural milieu can only help to further the effectiveness of treatment approaches depending on their diverse needs and experiences.

## 5. Influence of Cognitive and Emotional Factors on Pain Perception

The subjective experience of pain is influenced by many different cognitive and emotional factors. Cognitive factors, such as attention, anticipation, and evaluation of stimuli, play a vital role in modulating interpretation and response to pain sensations. For example, selective attention to pain-related stimuli can amplify the perceived intensity of pain, whereas distraction techniques can mitigate pain perception by diverting attention away from nociceptive stimuli [66]. However, the analgesic effect that various distractive methods have strongly depends on individual characteristics and the level of pain catastrophizing [67]. These results demonstrate the independent effect that catastrophizing has on pain intensity. Moreover, individuals with high levels of pain catastrophizing benefit less from distracting strategies [68], implying an involvement of a deeper maladaptive process. Overall, the interplay between cognitive and emotional factors is central to understanding individual differences in pain perception and coping. By addressing them, medical professionals can work on further improving treatment outcomes in patients experiencing pain, as well as enhance their quality of life.

The importance of cognitive factors and pain catastrophizing extends beyond individuals with chronic pain conditions to include research exploring various psychosomatic conditions. A cross-sectional study by Chen et al. (2022) [23] examined the relationship between psychosocial factors, maladaptive coping strategies, and pain intensity in patients suffering from irritable bowel syndrome (IBS). The strong correlation that exists between these variables suggests that an exaggerated state of interoception towards abdominal sensations in patients with IBS plays an important role in magnifying the symptoms, and further that by potentiating the negative cognitive thoughts through a maladaptive process such as catastrophizing, the pain intensity and disability related to it worsen. Overall, based on the extensive research on the association between pain catastrophizing and various psychosomatic disorders such as IBS, there is strong evidence to suggest that catastrophizing could prove to be an excellent target for further research into different treatment modalities. An especially interesting 2016 experimental study by Kjøgx et al. [69] demonstrated that by applying cognitive techniques, in this case by implementing hypnotic suggestions, levels of pain catastrophizing and pain intensity can be affected, both positively and negatively. Even though this study has significant limitations in terms of its sample size (44 participants), the possible clinical implications are significant. Not only does it support the claim that pain catastrophizing can be affected and thus treated, it also aligns with the cognitive-behavioral model of pain catastrophizing, reinforcing the idea that psychological and emotional factors are a cornerstone of chronic pain conditions.

Other important cognitive factors which influence pain intensity and pain-related disability are beliefs about the degree of control one has over the pain they experience, as well as the ability to predict and evade sensations of pain. Catastrophic interpretations of pain, exaggerated negative appraisals, and feelings of helplessness have been consistently associated with heightened pain intensity, emotional distress, and disability across various pain populations [61]. On the other hand, healthy cognitive coping strategies, such as positive reinterpretation and acceptance of pain, have been linked to lower pain severity and improved psychological well-being [28]. Another study that posited similar findings is a 2014 prospective cohort study by Pinto and associates [13]. Specifically, they investigated the relationship between psychosocial variables and postoperative surgical pain in patients undergoing abdominal hysterectomy and major joint arthroplasty. Their results showed that pain catastrophizing was the single most important predictor of acute post-surgery pain. Also, the study demonstrated that another important predicting factor for pain intensity after major joint arthroplasty is pre-surgery optimism. Similar results have been demonstrated in previous research as well [70], and for the concept of pain catastrophizing, they provide further proof of the clinically significant effect that healthy coping strategies and pleasant emotional states can have on pain-related outcomes.

Other emotional factors such as anxiety, depression, and fear play a significant role in shaping pain perception. A robust correlation has been consistently found between high levels of anxiety and depression and frequent exacerbations in pain intensity, as well as greater degree of disability [71]. Similarly, fear of pain that comes with movement, which can commonly be observed in individuals suffering from chronic pain conditions, can lead to avoidance behaviors, and resulting physical deconditioning, lessened quality of life, and ironically even higher levels of pain severity and pain-related distress [30,31].

Psychiatric comorbidities are common in patients with chronic pain syndromes, and among them the most common ones are anxiety and depression. Anxiety disorders, characterized by excessive worry, apprehension, and physiological arousal, are highly prevalent among individuals with chronic pain, with prevalence ranging from 20% to 50% [72,73]. Similarly, depression as a comorbidity in chronic pain patients has an estimated prevalence as high as 50% [74,75]. Both anxiety and depression have been consistently linked to heightened pain intensity, emotional distress, and disability, underscoring the bidirectional relationship between psychological and pain-related outcomes [74,75]. The underlying mechanisms of pain catastrophizing and psychiatric disorders such as depression and anxiety are overlapping, which may provide an explanation as to why their comorbidity is such a frequent occurrence. Specifically, anxiety has been shown to have an especially strong correlation to pain catastrophizing, generally, as well as independently with each subclass of magnification, rumination, and helplessness [24]. What is especially interesting is that even though the relationship between psychiatric disorders and pain intensity was until recently thought to be prevalently bidirectional, a recent prospective cohort study [25] conducted on a large sample found that an improvement in pain levels does not necessarily correlate to lower levels of depression and anxiety. These findings further suggest that underlying cognitive processes, which are a common finding in both psychiatric disorders and negative pain perception, especially pain catastrophizing, are perhaps an independent influencing factor which may not be affected by actual pain intensity levels. However, it should be noted that these findings might be skewed by the time period in which the study was conducted, specifically between 2015 and 2022, as it encompasses the period of the COVID pandemic which has resulted in abnormally high anxiety and depression levels [76,77].

The most critical aspect of the connection between pain catastrophizing and mental health disorders is its link with the risk of suicide. Patients suffering from chronic pain have alarmingly high rates of both suicidal ideation and suicide attempts [78]. Pain catastrophizing has been shown to significantly predict suicidal ideation and behavior in chronic pain patients with an opiate prescription [79], in patients with rheumatic disease [21], and in patients with chronic pain conditions in general [80]. A large study by Edwards et al. (2006) [81] determined that pain catastrophizing is the most important predictor of suicidal ideation along with the magnitude of depressive symptoms. All of these results suggest that there is a unique, independent association between pain catastrophizing and suicidal ideation which warrants further research, and signifies an important point clinicians should continuously have in mind when working with patients suffering from chronic pain.

## 6. Clinical Significance of Pain Catastrophizing

Numerous studies have explored the association between pain catastrophizing and pain intensity, and a robust positive correlation has been consistently found between the two (see Table 1).

For example, Craner et al. (2016) [16] examined the association between pain catastrophizing and pain intensity in a large sample of patients suffering from chronic pain. Their results demonstrated a significant bidirectional relationship between pain catastrophizing and depressed mood, decreased quality of life, and higher pain severity and life interference. Moreover, their findings imply that a multimodal rehabilitation can cause a significant decrease in pain catastrophizing, which further leads to a considerable improvement in treatment success. Some studies [90,91] have further found that in their complex dynamics, pain catastrophizing often precedes pain. These findings are in line with experimental studies which have found that changing the levels of pain catastrophizing in both patient and community-based population influences pain levels [69]. All of these findings suggest that there is reasonable hope that treatment strategies focusing on pain catastrophizing could be greatly beneficial to patients suffering from a variety of chronic pain syndromes.

Research also suggests that in terms of acute pain management, higher levels of pain catastrophizing are associated with poorer treatment outcomes and reduced responsiveness to analgesic medications [13]. Similarly, Quartana et al. [8] have found that preoperative pain catastrophizing predicted greater postoperative pain intensity and opioid consumption following surgery.

Similarly, in chronic pain management, pain catastrophizing has been linked to poorer treatment response in different therapeutic strategies including pharmacotherapy, physical therapy, and cognitive-behavioral therapy [28]. Similar findings have been produced in a meta-analysis by Burns et al. (2015) [92], which demonstrated that higher levels of pain catastrophizing were associated with lesser improvement in pain severity and physical function following treatment. Other studies with similar findings [3] have also shown an association between pain catastrophizing and other adverse outcomes such as pain-related negative affect, anxiety, depression, and disability.

The impact of pain catastrophizing on the success rates in treating pain further encompasses individuals’ psychosocial functioning and quality of life. Research has shown that individuals who exhibit higher levels of pain catastrophizing are more likely to experience persistent disability, psychological distress, and healthcare utilization, despite receiving standard treatments for chronic pain [8,28,92].

A major concern commonly associated with a poor treatment response is the risk of developing opioid use disorder among patients suffering from chronic pain. Studies have found that catastrophizing, anxiety, and depression are correlated with a higher risk for prescription opioid misuse [93,94]. Individuals who have high pain catastrophizing levels may be more likely to seek relief from opioids due to their perceived effectiveness in alleviating distressing symptoms. Moreover, as catastrophizing can impair coping mechanisms, individuals can begin to rely on opioids as a primary means of pain management, further increasing the risk of misuse and addiction.

Several risk factors contribute to pain catastrophizing and opioid misuse [95]. These include past experiences with pain or trauma, depression, and anxiety, as well as sociodemographic factors such as low socioeconomic status. Understanding these risk factors is crucial for developing targeted interventions for clinical use that address both pain catastrophizing and opioid misuse. One such intervention that has been found to successfully treat opioid misuse and craving in individuals with chronic pain is Mindfulness-Oriented Recovery Enhancement (MORE) [96,97]. It is a mindfulness-based training, and it includes several cognitive-behavioral therapy approaches that focus on the concurrent treatment of pain, cravings, and opioid misuse while reducing catastrophizing, anxiety, and depression. MORE is an important example of an intervention that, by adopting a broad personalized approach, addresses all of the important cognitive, emotional, and physical factors, which makes it very effective. Furthermore, a personalized approach to psychological treatments in pain management can reduce the dependence on pain control medication, and therefore the risk of opioid misuse [98]. Since this is a complex and multifaceted issue, in addition to an effective clinical approach, a comprehensive collaboration between healthcare providers and policymakers is essential. On a public health level, initiatives focusing on education, prevention, and access to non-opioid pain management alternatives are crucial. By addressing pain catastrophizing and promoting evidence-based interventions, healthcare systems can mitigate the risk of opioid misuse while improving outcomes for individuals living with chronic pain.

The integration of pain catastrophizing assessment tools into clinical decision-making processes has been garnering increasing attention in recent years. Several studies [8,92] have investigated the utility of these tools which could be used for purposes of identifying individuals at risk for adverse pain-related outcomes, which would enable an improvement in the selection of therapeutic interventions. Additionally, regular assessment of pain catastrophizing throughout the course of treatment could provide valuable information about treatment progress and the need for necessary adjustments to the management plan.

For instance, Quartana et al. [8] examined the predictive validity of the PCS in a sample of patients with chronic pain. The researchers found that higher levels of pain catastrophizing were associated with greater pain severity and disability, emphasizing the potential clinical utility of the PCS in identifying patients in need of more intensive pain management interventions.

Similarly, Suso-Ribera et al. (2017) [99] evaluated the effectiveness of pain catastrophizing assessment tools in predicting treatment outcomes. The findings revealed that pain catastrophizing was a significant predictor of treatment response, with higher levels of catastrophizing associated with poorer outcomes across diverse interventions.

Despite the potential benefits of using pain catastrophizing assessment tools in clinical practice, several challenges and limitations are noteworthy. As previously discussed, the complexity of pain catastrophizing poses a challenge when it comes to its measurement and interpretation. Furthermore, even though the PCS adequately represents the cognitive aspects of pain catastrophizing, it may not adequately demonstrate the affective and behavioral components [100] which are significant influencing factors to pain perception. Cultural and linguistic differences among a diverse and multiethnic patient population may also pose a limitation in adequately interpreting pain catastrophizing assessment instruments [65,101].

Moreover, the prevalent time constraints and resource limitations which occur in everyday clinical practice may impede the analysis of pain catastrophizing assessment results. Clinicians may prioritize other seemingly more pressing clinical concerns, such as pain severity and functional impairment, over the assessment of psychological factors. Furthermore, there is a significant lack of education of medical professionals when it comes to pain, its perception, catastrophizing, and maladaptive coping mechanisms in general [69]. This gap in knowledge combined with the routine use of pain catastrophizing assessment tools may result in a significant negative bias towards patients who score high on Pain Catastrophizing Scales, which can lead to patient mismanagement [9].

Despite these challenges, several interventions have been developed to target pain catastrophizing and improve pain management outcomes (see Table 2). Cognitive-behavioral therapy is one of the most widely studied and effective interventions for reducing pain catastrophizing and improving pain-related outcomes. Cognitive-behavioral therapy aims to identify and modify maladaptive cognitive and behavioral responses to pain through techniques such as cognitive restructuring, relaxation training, and behavioral activation. Numerous randomized controlled trials have demonstrated the efficacy of cognitive-behavioral therapy in reducing pain catastrophizing and pain severity, as well as improving psychological well-being and functional status across various pain conditions [31,102].

In addition, other interventions [103,104] targeting pain catastrophizing include mindfulness-based stress reduction (MBSR) [105], acceptance and commitment therapy (ACT) [106], and pain education programs [107]. These interventions emphasize acceptance, mindfulness, and self-regulation skills to promote adaptive coping with pain and reduce catastrophic thinking [103,104].

Understanding pain catastrophizing is a crucial step to developing personalized pain management strategies that address the specific needs of individuals and improve their pain outcomes. Recognizing pain catastrophizing allows healthcare providers to tailor interventions to address the specific needs of individuals, and tailored interventions have been found to successfully modulate and influence pain intensity levels [108].

Acknowledging the role pain catastrophizing plays while formulating a personalized pain management strategy is also important because it supports the necessity of a multidisciplinary approach, incorporating different medical, psychological, and lifestyle interventions [109]. While the term pain catastrophizing has been detrimental to some individuals suffering from chronic pain, and the need for its use has been debated [10], providing education about the concept and impact of pain catastrophizing, no matter the term used, can serve to empower individuals to participate in their pain management actively. Teaching coping skills and promoting self-efficacy can help individuals feel more in control of their pain and better equipped to manage it.

Overall, while challenges exist in measuring and interpreting pain catastrophizing in clinical practice, various cognitively-oriented interventions offer promising avenues for addressing maladaptive cognitive and emotional responses to pain and improving pain management outcomes.

**Table 2:** Summary of therapeutic interventions aimed at pain catastrophizing.

Intervention	Study	Condition	N	Findings
Acceptance and Commitment Therapy (ACT)	Lai et al. (2023) [110]	Chronic Pain	2293 ^1^	ACT showed significant effects on chronic pain especially when conducted face-to-face, and in the population with chronic headache and fibromyalgia. Considering the size and quality of the meta-analysis, it demonstrated considerable benefits in ACT for patients suffering from chronic pain.
Cognitive-Behavioral Therapy (CBT)	Alda et al. (2011) [111]	Fibromyalgia	169	CBT was superior to usual care in terms of function and quality of life, as well as in improving the level of pain catastrophizing and pain acceptance.
	Sun et al. (2020) [112]	Total Knee Replacement	80	In comparison to usual care, CBT was more efficient in reducing postoperative pain during activity from the fifth day to the third month, as well as reducing pain catastrophizing in the first three months.
	Buhrman et al. (2011) [113]	Chronic Back Pain	54	In the group of patients treated with CBT, statistically significant reductions were found in the level of pain catastrophizing and in the quality of life. The study demonstrated no other significant improvements when compared to other treatment. These findings suggest that internet-based CBT could have a supplementary role in chronic back pain treatment.
Mindfulness Based Stress Reduction (MBSR)	Garland et al. (2012) [114]	Irritable Bowel Syndrome	75	MBSR was found to significantly improve IBS symptoms, likely by modifying anxiety and catastrophizing related to abdominal sensations.
	La Cour et al. (2015) [115]	Chronic Pain	109	A significant effect of MBSR was found in the self-reported measure of health. Also, significant medium to large size effects were found for lower anxiety and depression, better mental quality of life, feeling in control of the pain, and higher pain acceptance. Small effect sizes were found for pain measures. These findings imply that a standardized MBSR can be clinically useful in management of patients with chronic pain.
	Cherkin et al. (2016) [116]	Back Pain	342	Treatment with MBSR or CBT, in comparison to usual standards of care, resulted in greater improvement in back pain as well as functional limitations, suggesting that it may be used as an effective therapy of chronic low back pain.
Pain Neuroscience Education (PNE)	Meeus et al. (2010) [117]	Chronic Fatigue Syndrome (CFS)	48	PNE was associated with an improved understanding of the neurophysiology of pain and a reduction of the ruminating subscale of the Pain Catastrophizing Scale, implying that PNE could be considered as a possible therapeutic modality in CFS and chronic pain.

^1^ This meta-analysis included 33 randomized controlled trials, with a total of 2293 participants.

## 7. Conclusions

The robust association between pain catastrophizing and adverse pain-related outcomes underscores the importance of assessing and addressing catastrophizing within the context of pain management interventions. While self-report measures such as the Pain Catastrophizing Scale have been proven as valuable tools for assessing pain catastrophizing in research and clinical settings, challenges remain when it comes to their integration into routine clinical practice. Despite these challenges, cognitively-oriented interventions targeting pain catastrophizing offer promising avenues for improving pain management outcomes through promoting adaptive coping strategies and enhancing patients’ ability to manage pain-related distress.

Moving forward, future research should continue to elaborate on the mechanisms that underlie pain catastrophizing and the role it plays in pain perception. Additionally, efforts should be made to advance the education of health care professionals on topics of pain catastrophizing and management in order to avoid negative biases. By improving our understanding of pain catastrophizing, researchers and clinicians can continue working on the quality of pain treatment and ultimately enhance the quality of life for individuals living with pain.

## Figures and Tables

**Table 1:** Summary of studies demonstrating a relationship of pain catastrophizing and pain-related clinical outcomes in various conditions.

Author (Year)	Condition	N	Country	Study Design	Findings
Belfer et al. (2013) [19]	Persistent Postmastectomy Pain (PPMP)	611	United States	Cross-Sectional	Catastrophizing was highly correlated with PPMP, especially within the first two years of surgery. Furthermore, catastrophizing along with anxiety, somatization, and sleep was associated with higher pain-related disability in patients.
Chen et al. (2022) [23]	Irritable Bowel Syndrome (IBS)	80	United States	Cross-Sectional	Psychosocial factors including catastrophizing, such as coping and self-efficacy, were strongly correlated with pain intensity and quality of life in young adults with IBS.
Craner et al. (2016) [16]	Chronic Pain Rehabilitation	648	United States	Within-Subject	There was a significant association between pain catastrophizing and decreased mental and physical health-related quality of life, higher pain severity, and life interference in patients with chronic pain. Moreover, participation in a comprehensive rehabilitation program significantly decreased pain catastrophizing in patients, which in turn positively affected treatment outcomes.
Crawford et al. (2021) [82]	Interstitial Cystitis/Bladder Pain Syndrome	226 ^1^	United States and Canada	Prospective Cohort	There was a temporal correlation between catastrophizing and pain, such that early changes in magnification predicted future changes in pain levels, and early changes in pain levels predicted future changes in rumination.
Doğru et al. (2018) [83]	Lumbopelvic Pain in Pregnancy	429	Turkey	Prospective Cohort	Pain catastrophizing fluctuated during the course of pregnancy, and was significantly correlated with depression, anxiety, physical and social functioning, and mental health.
Elvery et al. (2017) [17]	Chronic and Intermittent pain	260	Australia	Cross-Sectional	A significant correlation was found between pain catastrophizing and pain intensity, interference, and depression. Pain catastrophizing was shown to have the most robust association among patients with chronic and intermittent pain.
Flink et al. (2017) [18]	Vulvovaginal Pain	510	Sweden	Prospective Cohort	Catastrophizing was found to be a significant mediator between solicitous partner responses and pain.
Harris et al. (2017) [84]	Chronic Pain	436	United States	Cross-Sectional	Pain catastrophizing was related to spiritual distress directly, as well as through its relationship with depression. Pain interference was also associated with spiritual distress; however, only through its relationship with depression.
Harrison et al. (2015) [20]	Multiple Sclerosis Pain	612	United Kingdom	Cross-Sectional	Psychological factors, including catastrophizing, as well as distress, negative beliefs about pain, and avoidance of activity, were associated with worse pain intensity and outcomes.
Ikemoto et al. (2017) [61]	Knee Osteoarthritis	77	Japan	Cross-Sectional	In female patients with knee osteoarthritis, pain catastrophizing has been highly correlated to pain severity and quality of life.
Kjøgx et al. (2016) [69]	Chronic Headache	44	Denmark	Randomized Controlled Trial	Hypnotic suggestions significantly affected pain catastrophizing, as well as the levels of pain intensity and pain unpleasantness, with changes in pain catastrophizing being predictors of the changes in pain.
Lackner et al. (2005) [85]	Irritable Bowel Syndrome (IBS)	186	United States	Cross-Sectional	Worry was strongly associated with the emotionally unpleasant aspects of pain, especially suffering, and pain catastrophizing was found to be the mediating factor between the two.
Morasco et al. (2014) [22]	Hepatitis C	116	United States	Cross-Sectional	Pain catastrophizing and social support were associated with pain intensity, whereas age, pain severity, prescription opioid use, and chronic pain self-efficacy were associated with the level of pain interference.
Mortazavi Nasiri et al. (2017) [86]	Migraine	178	Iran	Descriptive-Correlational	There was a positive correlation between disability and catastrophizing, with pain intensity as a mediating factor. A high frequency of maladaptive coping strategies such as catastrophizing was found in the population of migraine patients with headache-related disability.
Newman et al. (2017) [87]	Chronic Pain	290	United States	Cross-Sectional	Depression and catastrophizing were mediating factors for age and various pain outcomes, and catastrophizing mediated the effects of literacy and poverty.
Novak et al. (2011) [12]	Peripheral Neve Injury	158	Canada	Cross-Sectional	Disability due to pain was predicted by various factors including pain catastrophizing.
Noyman-Veksler et al. (2017) [80]	Chronic Pain	428/165 ^2^	Israel	Cross-Sectional	Pain catastrophizing was predictive of pain, and distress was predictive of pain-related disability. Pain catastrophizing was also associated with depression and suicidal ideation.
Park et al. (2016) [88]	Chronic Musculoskeletal Pain	357	Korea	Cross-Sectional	Pain catastrophizing, as well as older age and insomnia, were significantly associated with higher pain levels
Pinto et al. (2014) [13]	Acute Postoperative Pain	252	Portugal	Prospective Cohort	Pain catastrophizing was found to be a significant predictor for acute postoperative pain.
Shelby et al. (2009) [14]	Noncardiac Chest Pain	97	United States	Cross-Sectional	Through relationship with pain catastrophizing, chest pain and anxiety were found to indirectly influence physical disability. Chest pain also showed a significant indirect relationship with psychosocial disability through pain catastrophizing.
Shim et al. (2017) [21]	Rheumatic Disease	360	Korea	Cross-Sectional	Magnification element of pain catastrophizing had a significant indirect relationship with suicide risk through perceived burdensomeness, depression, and perceived social support.
Shim et al. (2018) [15]	Headache	247	Korea	Cross-Sectional	Pain catastrophizing was found to be the only significant mediator through which alexithymia was associated with headache-related outcomes.
Taylor et al. (2017) [89]	Fibromyalgia	220	United States	Within-Subject	Catastrophizing and coping efficacy were associated with end-of-day pain in patients with fibromyalgia.

^1^ Baseline number of participants was 226, while on the two follow-up surveys it was 183 and 151 respectively; ^2^ Results of two studies are discussed jointly in this paper; there were 428 participants in study 1, and 165 participants in study 2.
